# Anti-inflammatory evaluation of the methanolic extract of *Taraxacum officinale* in LPS-stimulated human umbilical vein endothelial cells

**DOI:** 10.1186/s12906-017-2022-7

**Published:** 2017-11-29

**Authors:** Daun Jeon, Seok Joong Kim, Hong Seok Kim

**Affiliations:** 1 Department of Molecular Medicine, College of Medicine, Inha University, Incheon, 22212 Republic of Korea; 2 Hypoxia-related Disease Research Center, College of Medicine, Inha University, Incheon, 22212 Republic of Korea; 3 Department of Food and Nutrition, Dongduk Women’s University, Seoul, 02748 Republic of Korea

**Keywords:** *Taraxacum officinale*, Anti-inflammation, Endothelial cell, Endotoxin, NF-κb

## Abstract

**Background:**

Atherosclerosis is a chronic vascular inflammatory disease. Since even low-level endotoxemia constitutes a powerful and independent risk factor for the development of atherosclerosis, it is important to find therapies directed against the vascular effects of endotoxin to prevent atherosclerosis. *Taraxacum officinale* (TO) is used for medicinal purposes because of its choleretic, diuretic, antioxidative, anti-inflammatory, and anti-carcinogenic properties, but its anti-inflammatory effect on endothelial cells has not been established.

**Methods:**

We evaluated the anti-inflammatory activity of TO filtered methanol extracts in LPS-stimulated human umbilical vein endothelial cells (HUVECs) by monocyte adhesion and western blot assays. HUVECs were pretreated with 100 μg/ml TO for 1 h and then incubated with 1 μg/ml LPS for 24 h. The mRNA and protein expression levels of the targets (pro-inflammatory cytokines and adhesion molecules) were analyzed by real-time PCR and western blot assays. We also preformed HPLC analysis to identify the components of the TO methanol extract.

**Results:**

The TO filtered methanol extracts dramatically inhibited LPS-induced endothelial cell–monocyte interactions by reducing vascular cell adhesion molecule-1 and monocyte chemoattractant protein-1, and pro-inflammatory cytokine expression. TO suppressed the LPS-induced nuclear translocation of NF-κB, whereas it did not affect MAPK activation.

**Conclusions:**

Our findings demonstrated that methanol extracts of TO could attenuate LPS-induced endothelial cell activation by inhibiting the NF-κB pathway. These results indicate the potential clinical benefits and applications of TO for the prevention of vascular inflammation and atherosclerosis.

**Electronic supplementary material:**

The online version of this article (10.1186/s12906-017-2022-7) contains supplementary material, which is available to authorized users.

## Background

Insults to the vasculature can cause a wide range of life-threatening diseases, including stroke, myocardial infarction, hypertension, and chronic kidney disease. Inflammation is emerging as a key contributor to many vascular diseases, and furthermore plays a major role in autoimmune diseases, arthritis, allergic reactions, and cancer.

Besides autoimmune disorders related to vascular inflammation, a more common chronic vascular inflammatory disease is atherosclerosis [1, 2]. Clinical evidence indicates that atherosclerosis is accelerated in patients suffering from large and medium-sized vessel vasculitis [3].

Endotoxemia, the presence of endotoxin in the blood, constitutes a strong risk factor for early atherogenesis in subjects with chronic or recurrent bacterial infections [4]. Even low-level endotoxemia (as low as 50 pg/ml) can be a powerful and independent risk factor for the development of atherosclerosis [5] through multiple mechanisms, including increases in reactive oxygen species, chemotactic and pro-inflammatory cytokines, and adhesion molecules. Therefore, it is important to find therapies directed against the vascular effects of endotoxin to prevent atherosclerosis in humans.

Most therapy regimens for treating atherosclerosis aim at modulating hypertension and hyperlipidemia or controlling hemostasis in order to avoid thrombotic complications [6]. However, many of these modalities often neglect the role of inflammation in atherosclerosis [6]. As these therapies remain insufficient in reducing the burden of atherosclerosis-related mortality, other approaches like complementary and alternative medicine have recently started to gain more attention [7, 8].

*Taraxacum officinale* (TO), commonly known as dandelion, is used for medicinal purposes because of its choleretic, diuretic, antioxidative, anti-inflammatory, and anti-carcinogenic properties [9, 10]. The anti-inflammatory effects of extracts of TO or its single components have been reported in both in vitro and animal models. TO extracts (100 and 1000 μg/ml) were demonstrated to inhibit lipopolysaccharide (LPS)-induced tumor necrosis factor-alpha (TNF-α) production in rat astrocytes by inhibiting interleukin-1 (IL-1) production [11]. Luteolin and luteolin-7-*O*-glucoside, two active components from TO flower extracts, significantly suppressed the production of both inducible nitric oxide synthase and cyclooxygenase-2 in LPS-activated mouse macrophage RAW264.7 cells [12]. Pretreatment with TO extracts also protected against LPS-induced acute lung injury in mice [13]. However, the effect of TO on endothelial activation has not been established.

Our study shows that TO reduces the expression of vascular cell adhesion molecule-1 (VCAM-1) and pro-inflammatory cytokines in endothelial cells, and decreases mononuclear cell adhesion by suppressing nuclear factor-kappa B (NF-κB) signaling.

## Methods

### Reagents

LPS, dimethyl sulfoxide (DMSO), and Hoechst 33,258 were purchased from Sigma-Aldrich (St. Louis, MO, USA). Calcein AM was obtained from Thermo Fisher Scientific (Waltham, MA, USA). Antibodies against VCAM-1, intercellular adhesion molecule-1 (ICAM-1), β-actin, NF-κB p65, inhibitor of NF-κB alpha (IκBα), and phospho-IκBα were obtained from Cell Signaling Technology (Danvers, MA, USA).

### Methanol extracts of *Taraxacum officinale*

Methanol extracts (code numbers: PB5027.2) from whole plants of TO were purchased from the Plant Extract Bank at the Korea Research Institute of Bioscience and Biotechnology (Daejeon, Republic of Korea; http://extract.kribb.re.kr; E-mail: plantext@kribb.re.kr) [14, 15]. The extracts were dissolved in DMSO at a concentration of 100 mg/ml.

### Cell culture

Human umbilical vein endothelial cells (HUVECs) were obtained from ScienCell Research Laboratories (San Diego, CA, USA) [16]. The cells were cultured in endothelial cell medium (ScienCell Research Laboratories) containing 5% (*v*/v) fetal bovine serum, at 37 °C under an atmosphere with 5% (v/v) CO_2_ and 95% humidity.

### Cytotoxicity assay

Cell viability was assessed by using the CellTilter 96 Aqueous One Solution Cell Proliferation Assay (Promega Corporation, Madison, WI, USA), according to the manufacturer’s instructions. Cells were seeded at a density of 1 × 10^4^ cells/well into 96-well plates. After their subjection to different treatments, the cells were incubated with 3-(4,5-dimethylthiazol-2-yl)-5-(3-carboxymethoxyphenyl)-2-(4-sulfophenyl)-2H–tetrazolium, inner salt (MTS) solution at a final concentration of 0.4 mg/ml for 4 h at 37 °C. One Solution Reagent was then added directly to the culture wells, and the plates were incubated for 4 h, following which the absorbance at 490 nm was recorded with a 96-well plate reader [17, 18]. The absorbance was measured at 490 nm with a Multiskan GO microplate spectrophotometer (Thermo Fisher Scientific).

### Western blot analysis

Cells were washed with ice-cold PBS and lysed on ice in RIPA lysis buffer (50 mM Tris-HCl (pH 7.5), 150 mM NaCl, 1% Nonidet P-40, 0.1% sodium dodecyl sulfate, and 0.5% sodium deoxycholate) supplemented with protease and phosphatase inhibitors. Aliquots with equal amounts of protein were loaded and separated on a SDS-PAGE gel. The proteins on the gel were then transferred to a nitrocellulose membrane (Bio-Rad, Hercules, CA, USA) and probed using specific antibodies as indicated. The bands were detected by chemiluminescence on a ChemiDoc imaging system (Bio-Rad). To control for sample loading, the blots were subsequently stripped and re-probed for total IκBα or β-actin.

### RNA extraction and real-time reverse-transcription polymerase chain reaction

Total RNA was isolated from the cells, using the NucleoSpin RNA Plus Kit (MACHEREY-NAGEL, Düren, Germany), and 1 μg was then used for cDNA synthesis with the iScript cDNA Synthesis Kit (Bio-Rad). The resulting cDNA was PCR amplified with the appropriate primer pairs: *MCP-1*, 5′-AGAATCACCAGCAGCAAGTGTCC-3′ (forward) and 5′-TCCTGAACCCACTTCTGCTTGG-3′ (reverse); *TNF-α*, 5′-CTCTTCTGCCTGCTGCACTTTG-3′ (forward) and 5′-ATGGGCTACAGGCTTGTCACTC-3′ (reverse); *IL-1β*, 5′- CCACAGACCTTCCAGGAGAATG-3′ (forward) and 5′-GTGCAGTTCAGTGATCGTACAGG-3′ (reverse); *IL-6*, 5′-AGACAGCCACTCACCTCTTCAG-3′ (forward) and 5′- TTCTGCCAGTGCCTCTTTGCTG-3′ (reverse); and *18S rRNA*, 5′-AACCCGTTGAACCCCATT-3′ (forward) and 5′-CCATCCAATCGGTAGTAGCG-3′ (reverse) (Bioneer, Daejeon, Republic of Korea). The RT-qPCR was performed and analyzed with a CFX Connect Real-Time PCR detection system (Bio-Rad), and the gene expression levels were normalized to that of 18S rRNA as the housekeeping gene [19].

### Analysis of monocyte adhesion to HUVECs

HUVECs were seeded and incubated in 6-well plates until they reached >85% confluence. Subsequently, the cells were pre-incubated with the TO extract (100 μg/ml) for 1 h prior to stimulation with LPS (1 μg/ml) for 24 h. Human monocytic THP-1 cells were labeled with 5 μM Calcein AM for 30 min in RPMI-1640 medium and then added to the HUVEC-containing 6-well plates and incubated for 1 h. Subsequently, unbound monocytes were removed by 3 washes with warm phosphate-buffered saline (PBS) [20]. Bound monocytes were determined using a fluorescence microscope (EVOS FL Cell Imaging System, Thermo Fisher Scientific).

### Immunofluorescence analysis of NF-κB p65 nuclear translocation

HUVECs were pre-incubated with the TO extract (100 μg/ml) for 1 h, followed by LPS (1 μg/ml) for 1 h. The cells were then fixed with 4% paraformaldehyde in PBS (pH 7.4) for 20 min at room temperature, and permeabilized with 0.1% Triton X-100. Nonspecific binding was blocked with 5% normal goat serum for 1 h. Then, the cells were incubated overnight with NF-κB p65 antibody at 4 °C, followed by incubation with Alexa Fluor 594-conjugated goat anti-rabbit IgG (Thermo Fisher Scientific) for 1 h at room temperature. Nuclei were stained with Hoechst 33,258. The locations of NF-κB p65 and nuclei were determined using a fluorescence microscope (EVOS FL Cell Imaging System). Results were expressed as the percentage of NF-κB p65-positive cells in the total cells counted from 4 randomly chosen high-power (×20) fields in each well. Each assay was performed in triplicate.

### High-performance liquid chromatography analysis

The TO methanol extracts were filtered (Millipore 0.45 μm) and 5 μl was injected into a high-performance liquid chromatography (HPLC) column (Supelcosil LC-18, 25 cm × 4.6 mm × 5 μm), at 25 °C. The extract separation was carried out on an HPLC device composed of Waters 510 pumps, 2489 UV/Vis detector, gradient controller, and Rheodyne injector, with a solvent A and B mixture as follows: methanol:acetic acid:water (10:2:88; solvent A) and methanol:acetic acid:water (90:3:7; solvent B) at a flow of 1 ml/min, with detection at 280 nm. The solvent A/B gradient applied was as follows: A from 100% to 85% (minutes 0–10), A from 85% to 50% (minutes 10–30), A from 50% to 15% (minutes 30–45), and A from 15% to 100% (minutes 45–55) [21]. The identification of peaks was made by comparison with HPLC chromatograms of individual pure phenolic acid (gallic, protocatechuic, chlorogenic, caffeic, *p*-coumaric, and ferulic acids) standards procured from Sigma-Aldrich.

### Statistics

Data were analyzed by analysis of variance (Sigma Stat 12.0) and tested for use of parametric or nonparametric post hoc analysis. Multiple comparisons were performed using the least significant difference method. All data are presented as the mean ± standard error of at least 3 independent experiments. Results were considered statistically significant at the *p <* 0.05 level.

## Results

### Effect of TO on HUVEC viability

**Fig. 1:**
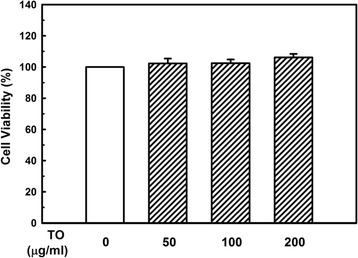
Effect of the *Taraxacum officinale* (TO) methanol extract on the viability of HUVECs. Cells were treated with different concentrations of the TO extract for 24 h, and viability was determined by the MTS assay. The results of independent experiments were averaged as the percentage cell viability compared with untreated control. Results shown are the mean ± SE of triple determinations

The cytotoxicity of the TO extract was examined by MTS assay. The viability of HUVECs treated with TO for 24 h was not affected at any of the extract concentrations (50, 100, and 200 μg/ml) tested (Fig. 1).

### Effect of TO on monocyte adhesion to LPS-stimulated endothelial cells

**Fig. 2:**
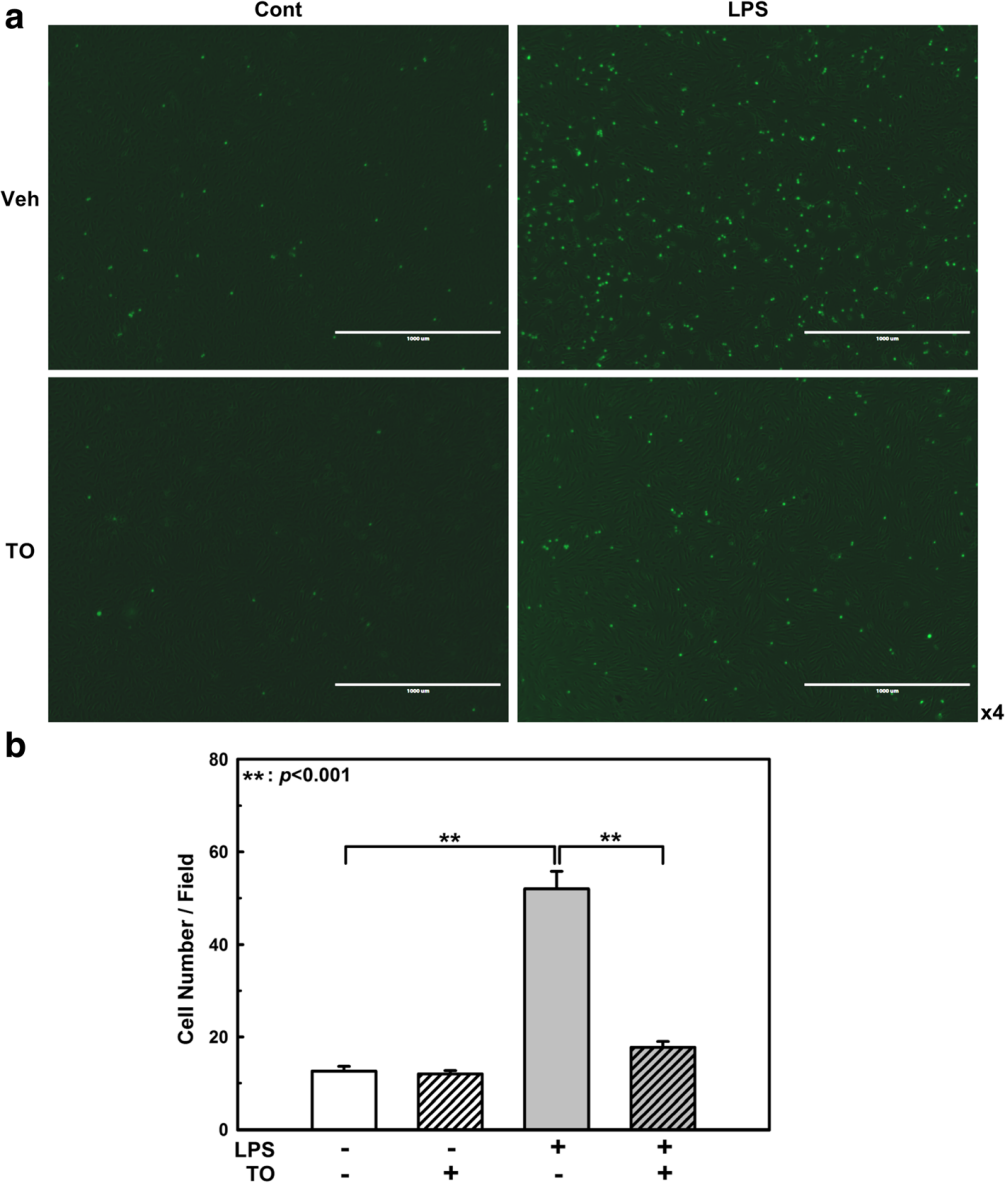
Effect of the *Taraxacum officinale* (TO) methanol extract on LPS-induced monocyte adhesion to HUVECs. HUVECs were pretreated with TO extract (100 μg/ml) for 1 h, and then stimulated with 1 μg/ml LPS for 24 h. Adhesion of fluorescent THP-1 monocytes was photographed by fluorescence microscopy (**a**) and analyzed (**b**). Scale bars: 1000 μm. Results shown are the mean ± SE of 3 independent experiments. **p < 0.01 vs LPS only treatment

To determine the effect of TO on endothelial cell–monocyte interactions, we performed adhesion assays. We used human monocytic THP-1 cells to mimic the interaction of monocytes with the endothelium. LPS treatment increased THP-1 cell adherence to the endothelial surface. Pretreatment with the TO extract (100 μg/ml, 1 h) decreased the LPS-induced THP-1 cell adhesion to a near-baseline level, indicating that TO can significantly decrease LPS-induced monocyte adhesion to endothelial cells (Fig. 2).

### Effect of TO on adhesion molecule and inflammatory cytokine expression in LPS-stimulated endothelial cells

**Fig. 3:**
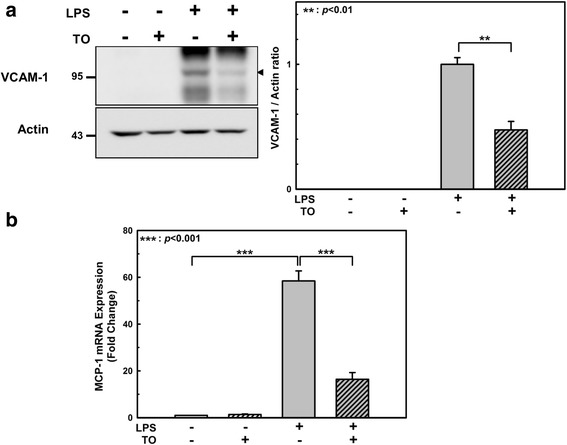
Effect of the *Taraxacum officinale* (TO) methanol extract on LPS-induced VCAM-1 and MCP-1 expression. HUVECs were pretreated with TO extract (100 μg/ml) for 1 h and then incubated with 1 μg/ml LPS for 24 h. **a** Total cell lysate was prepared, the proteins were separated by SDS-PAGE, and the VCAM-1 levels were assessed by western blot analysis. **b** The mRNA levels were normalized to that of a housekeeping gene (18S rRNA) as well as the vehicle-only-treated control, and the 2^-ΔΔCt^ for each mRNA is reported. Results shown are the mean ± SE of 3 independent experiments. **p < 0.01, ***p < 0.001 vs LPS only treatment

Endothelial activation involves the coordinated induction of genes encoding for leukocyte adhesion molecules (e.g., VCAM-1 and ICAM-1), chemotactic factors (e.g., monocyte chemoattractant protein-1 [MCP-1]), and growth factors (e.g., macrophage colony-stimulating factor). Endothelial VCAM-1 is an important mediator of mononuclear cell adhesion, given that its cognate ligand, the integrin very late antigen-4, is selectively expressed on monocytes (and on some T lymphocytes) but not on neutrophils [22]. We therefore examined whether TO treatment decreases VCAM-1 induction in response to LPS stimulation in HUVECs. Pretreatment of TO decreased the LPS-induced VCAM-1 expression by 53% (Fig. 3a). The potency of 100 μg/ml TO was quite compatible with that of 1 mM acetylsalicylic acid (Additional file 1: Figure S1). We also measured the ICAM-1 protein levels but did not observe any changes with TO treatment (Additional file 1: Figure S2).

Since MCP-1 is one of the key chemokines that regulate leukocyte trafficking and modulate interactions between leukocytes and endothelial cells [23, 24], we next examined whether TO inhibits MCP-1 expression in LPS-stimulated HUVECs. To this end, we used RT-qPCR to examine MCP-1 mRNA expression in HUVECs in response to LPS stimulation. MCP-1 mRNA expression was induced by LPS (58.5-fold), but this effect was significantly decreased (16.4-fold) in HUVECs pretreated with the TO extract (Fig. 3b).

**Fig. 4:**
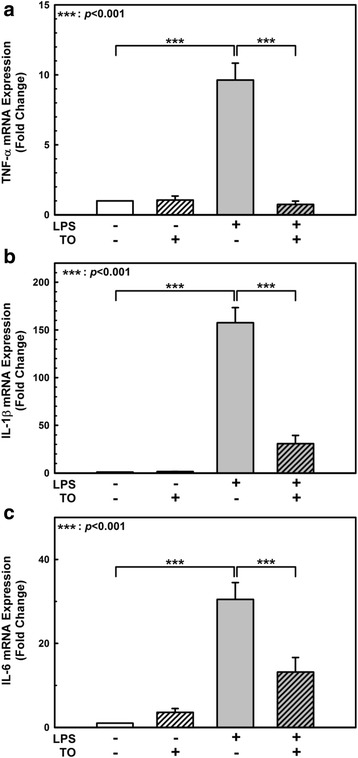
Effect of the *Taraxacum officinale* (TO) methanol extract on LPS-induced pro-inflammatory cytokine expression. The pro-inflammatory cytokine mRNA levels were normalized to that of a housekeeping gene (18S rRNA) as well as the vehicle-only-treated control, and the 2^-ΔΔCt^ for each mRNA is reported. Results shown are the mean ± SE of 3 independent experiments (**a**, **b**, **c**). ***p < 0.001 vs LPS only treatment

We also measured pro-inflammatory cytokine mRNA expression levels by RT-qPCR. TNF-α, IL-1β, and IL-6 were induced by 9-, 157-, and 30-fold, respectively (Fig. 4). However, these pro-inflammatory cytokine mRNA expression levels were markedly reduced by TO pretreatment (Fig. 4).

### Effect of TO on NF-κB activation in LPS-stimulated endothelial cells

LPS stimulation can lead to the activation of several intracellular signaling molecules. It has been reported that LPS can activate the mitogen-activated protein kinase (MAPK) [25, 26] and NF-κB pathways in endothelial cells [27]. These pathways are involved in controlling the expression of adhesion molecules and pro-inflammatory cytokines. Therefore, we first examined whether the TO extract inhibits MAPK (Erk1/2, p38MAPK, and JNK) activation in response to LPS stimulation. Western blot analysis indicated that TO did not markedly affect LPS-induced MAPK activation (Additional file 1: Figure S3).

**Fig. 5:**
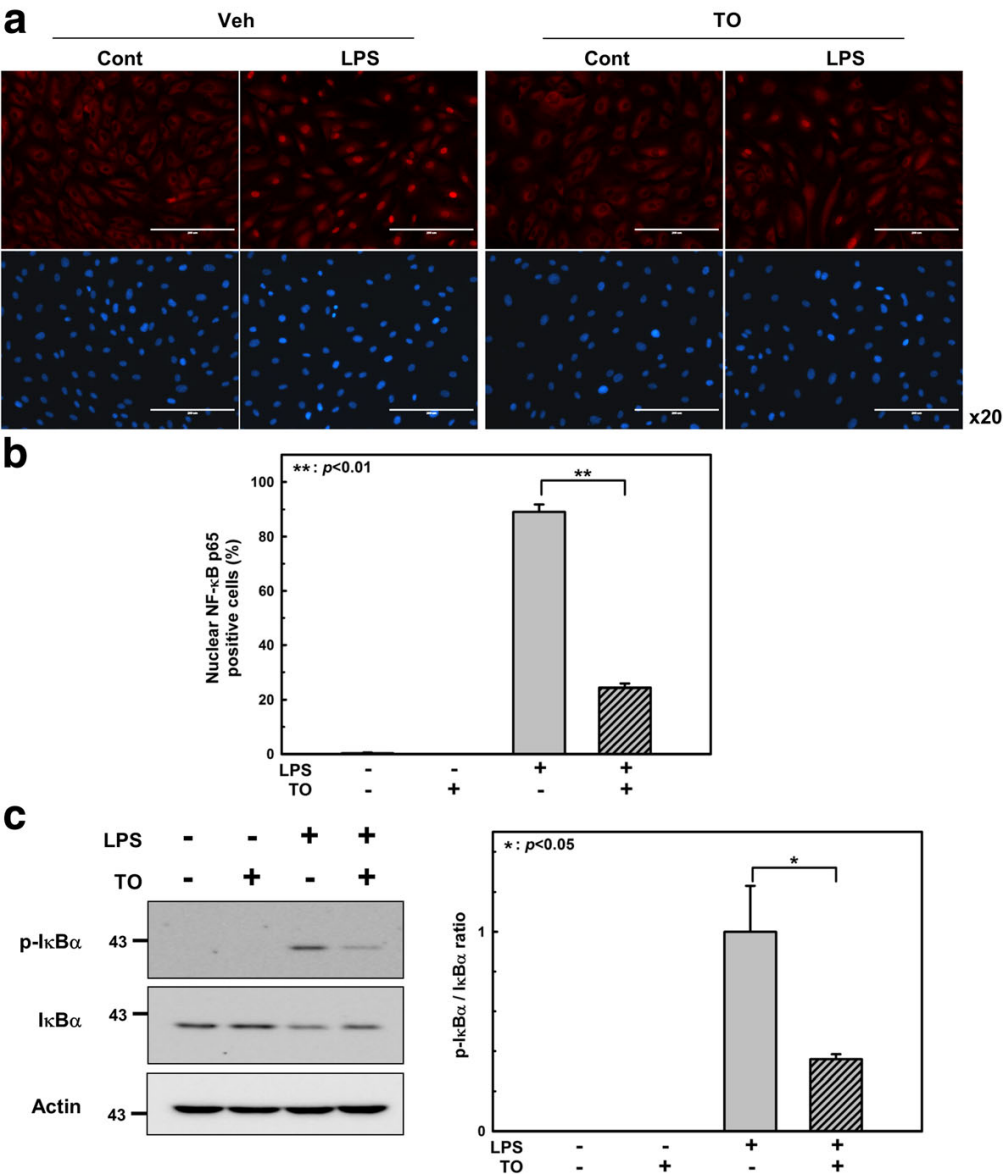
Effect of the *Taraxacum officinale* (TO) methanol extract on the LPS-induced nuclear translocation of NF-κB p65 in HUVECs. HUVECs were pretreated with TO extract (100 μg/ml) for 1 h and then stimulated with LPS (1 μg/ml) for 1 h. **a** Representative images of immunofluorescence staining showing NF-κB p65 (red) and cell nuclei stained with Hoechst 33,258 (blue). Scale bars: 200 μm. **b** Quantitation of NF-κB p65 nuclear translocation in the indicated groups. **c** IκBα phosphorylation assessed by western blot analysis. Results are shown as the mean ± SE (*n* = 3–4). *p < 0.05, **p < 0.01 vs LPS only treatment

To determine whether TO regulates VCAM-1 and pro-inflammatory cytokine expression by inhibiting NF-κB, we performed immunofluorescence staining of the NF-κB p65 subunit and showed that TO markedly suppresses LPS-induced NF-κB nuclear translocation (Fig. 5a and b). Since NF-κB nuclear translocation is regulated by IκBα, the degradation of which is triggered by phosphorylation, we studied whether TO treatment was associated with the inhibition of IκBα phosphorylation. As shown in Fig. 5c, TO pretreatment inhibited IκBα phosphorylation by 65%, and the degradation of IκBα induced by LPS in 30 min was also partially recovered by TO treatment. These results suggest that TO reduces the LPS-induced endothelial expression of VCAM-1 and pro-inflammatory cytokines by suppressing activation of the NF-κB pathways.

### HPLC analysis of the TO methanol extract

**Fig. 6:**
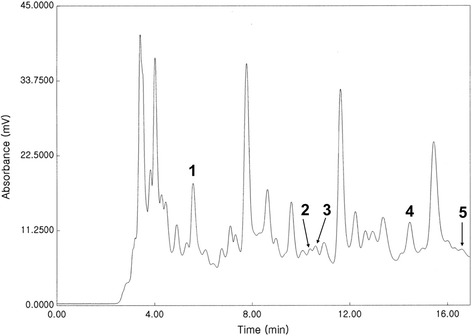
HPLC chromatogram of the *Taraxacum officinale* methanol extract. 1. Protocatechuic acid; 2. Chlorogenic acid; 3. Caffeic acid; 4. *p*-Coumaric acid; and 5. Ferulic acid. The other peaks were not identified owing to a lack of authentic compounds to compare with

Several peaks were monitored in the HPLC profile of the TO extract (Fig. 6). By comparing the chromatographic peaks of TO with those of the reference standards, protocatechuic acid, chlorogenic acid, caffeic acid, *p*-coumaric acid, and ferulic acid were qualitatively identified in the extract. The other peaks of the extract could not be identified owing to a lack of authentic references to compare with.

## Discussion

Inflammation is a set of interrelated processes in response to injuries caused by a variety of biological, chemical, and physical stimuli [28]. Vascular endothelial cells form an interface between blood flow and the vessel wall, and execute a number of important functions in the maintenance of the body’s homeostasis [29]. Not only does the endothelium provide a nonadhesive and highly selective physical barrier to control the vascular permeability, but it also secretes a large number of vasoactive substances to regulate the vascular tone and remodeling of the vessel wall [30]. Most importantly, as the key regulators and major targets of the inflammatory process, endothelial cells are indispensable components of inflammation. Endothelial cells are constantly exposed to various biological, chemical, and mechanical milieus, and maintain a quiescent state with antithrombotic, anti-inflammatory, and antiproliferative properties [31]. During inflammatory responses, endothelial cells are phenotypically converted into an “activated” state that is characterized by increased permeability, induced leukocyte adhesion, and gene expression of a variety of pro-inflammatory cytokines [32]. Endothelial cell activation leads to endothelial dysfunction, which can be caused by several conditions, including various infections, diabetes or the metabolic syndrome, hypertension, smoking, and physical inactivity [33, 34].

Atherosclerosis is a chronic inflammatory disease characterized by monocyte infiltration and macrophage accumulation in the vessel wall [35]. One potentially important source of inflammation is endotoxin (LPS), a unique glycolipid that comprises most of the outer leaflet of the outer wall of gram-negative bacteria [36, 37]. The Bruneck study provided the first epidemiological evidence that subclinical endotoxemia constitutes a strong risk factor for the development of carotid atherosclerosis, particularly among smokers [5]. A 5-year prospective study showed that in subjects without atherosclerosis at baseline, ~40% of newly developed carotid atherosclerosis was attributable to chronic infection, making it a leading atherogenic risk predictor [4]. Moreover, chronic infections caused by gram-negative bacteria conferred an increased risk of atherosclerosis development, even in low-risk subjects who lacked conventional vascular risk factors [4]. Even if there is no apparent infection source, a high-fat diet augments plasma LPS (“metabolic endotoxemia”) to a concentration sufficient to trigger inflammation and metabolic diseases, such as obesity and diabetes [38, 39], by increased intestinal permeability, favoring translocation of microbiome-derived LPS to the bloodstream [38]. In addition, the plasma LPS level is also markedly increased in diabetic patients compared with that in non-diabetic subjects [40, 41]. These observations support the hypothesis that chronic exposure to endotoxins may be pathogenically linked to atherosclerosis.

TO has long been used as a herbal remedy to treat medical problems, including inflammatory disease [10, 42–44]. The anti-inflammatory effects of TO extracts or its single components have been reported in both in vitro and animal models [11, 13, 45–47]. Among the identified components in our HPLC analysis, protocatechuic acid [48, 49], chlorogenic acid [50], caffeic acid [51, 52], and ferulic acid [53, 54] have showed anti-inflammatory activity in the endothelial system. Recently, Hu et al. reported that aqueous extracts of TO inhibited both TNF-α and ICAM-1 expression in LPS-stimulated rat mammary microvascular endothelial cells [55]. However, the authors did not identify any underlying molecular mechanisms of the TO extracts.

In this study, we have shown that the anti-inflammatory effect of the TO methanol extract on human endothelial cells is mediated through its reduction of VCAM-1 and pro-inflammatory cytokine expression. Since endothelial VCAM-1 is an important mediator of mononuclear cell (monocytes and some T lymphocytes) adhesion, our finding of the reduced VCAM-1 expression explains the significantly inhibited monocyte adhesion to LPS-stimulated endothelial cells. We also examined whether TO reduces ICAM-1 induction in LPS-stimulated endothelial cells, but could not repeat the findings of Hu et al. [55] in this study. This might be due to the difference in solvents used for the TO extraction, because the solvent type is one of the most common factors affecting bioactive compounds in extraction processes [56, 57]. Since acetylsalicylic acid, also known as Aspirin, is reported to suppress endothelial VCAM-1 induction [58], we compared the potency of TO with acetylsalicylic acid on VCAM-1 induction in LPS-stimulated HUVECs (Additional file 1: Figure S1). At 1 mM, acetylsalicylic acid was significantly more potent than 100 μg/ml of TO at suppressing LPS-stimulated VCAM-1 induction, although 500 μM of acetylsalicylic acid only inhibited VCAM-1 induction by 18% (data not shown).

To elucidate the underlying molecular mechanism of the TO effect, components of the MAPK signaling pathway and NF-κB and its upstream effectors were examined, because this pathway and transcription factor play an essential role in the modulation of LPS-induced inflammation and transcriptional regulation. As shown in Fig. 5, the phosphorylation of IκBα and the nuclear translocation of p65 were suppressed by TO pretreatment. However, TO had no apparent effect on the phosphorylation of Erk1/2, p38MAPK, and JNK (Additional file 1: Figure S3). These results suggest that inhibition of the LPS-induced transactivation of p65 consequently reduces the expression of inflammatory mediators in TO-pretreated HUVECs.

## Conclusion

Our findings demonstrated that the methanol extract of TO could attenuate LPS-induced endothelial activation by inhibiting the NF-κB pathway. Although TO has been used in the treatment of various disease, recent studies suggest it can be used effectively in the treatment of endothelial inflammation and atherosclerosis. However, to confirm these claims, more systematic, well-designed animal and randomized clinical studies with sufficient sample sizes are essential to investigate the exact action mechanisms, safety, and pharmacokinetics of this plant.

## Additional file

Additional file 1: Figure S1. Comparison of TO efficacy with acetylsalicylic acid (ASA). HUVECs were pretreated with TO (100 μg/ml) or different concentrations of ASA for one hour, and then stimulated with 1 μg/ml LPS for 24 h. VCAM-1 and actin levels were assessed by Western blot analysis. Arrow head indicates VCAM-1. **Figure S2.** TO decreases VCAM-1 induction dose dependently but does not affect ICAM-1 levels in LPS-stimulated HUVECs. HUVECs were pretreated with different concentrations of TO for one hour, and then stimulated with 1 μg/ml LPS for 24 h. VCAM-1, ICAM-1, and actin levels were assessed by Western blot analysis. Arrow head indicates VCAM-1. **Figure S3.** TO does not affect MAPK activation induced by LPS stimulation in HUVECs. HUVECs were preincubated with TO (100 μg/ml) for one hour and then stimulated with LPS (1 μg/ml) for 30 min. MAPKs activation was assessed by Western blot analysis. (PDF 368 kb)

## Abbreviations

DMSO: Dimethyl sulfoxide
HPLC: High-performance liquid chromatography
HUVEC: Human umbilical vein endothelial cell
IκBα: Inhibitor of NF-κB alpha
ICAM-1: Intercellular adhesion molecule-1
IL-1: Interleukin-1
LPS: Lipopolysaccharide
MAPK: Mitogen-activated protein kinase
MCP-1: Monocyte chemoattractant protein-1
NF-κB: Nuclear factor-kappa B
TNF-α: Tumor necrosis factor-alpha
TO: *Taraxacum officinale*
VCAM-1: Vascular cell adhesion molecule-1
