# Supplementary material for: Anti-inflammatory evaluation of the methanolic extract of Taraxacum officinale in LPS-stimulated human umbilical vein endothelial cells
Source: BMC Complement Altern Med. 2017 Nov 29;17:508. doi: 10.1186/s12906-017-2022-7 (PMC5707789; doi:10.1186/s12906-017-2022-7)
Supplement: Additional file 1: Figure S1. — Comparison of TO efficacy with acetylsalicylic acid (ASA). HUVECs were pretreated with TO (100 μg/ml) or different concentrations of ASA for one hour, and then stimulated with 1 μg/ml LPS for 24 h. VCAM-1 and actin levels were assessed by Western blot analysis. Arrow head indicates VCAM-1. Figure S2. TO decreases VCAM-1 induction dose dependently but does not affect ICAM-1 levels in LPS-stimulated HUVECs. HUVECs were pretreated with different concentrations of TO for one hour, and then stimulated with 1 μg/ml LPS for 24 h. VCAM-1, ICAM-1, and actin levels were assessed by Western blot analysis. Arrow head indicates VCAM-1. Figure S3. TO does not affect MAPK activation induced by LPS stimulation in HUVECs. HUVECs were preincubated with TO (100 μg/ml) for one hour and then stimulated with LPS (1 μg/ml) for 30 min. MAPKs activation was assessed by Western blot analysis. (PDF 368 kb) [file 12906_2017_2022_MOESM1_ESM.pdf]

Additional files

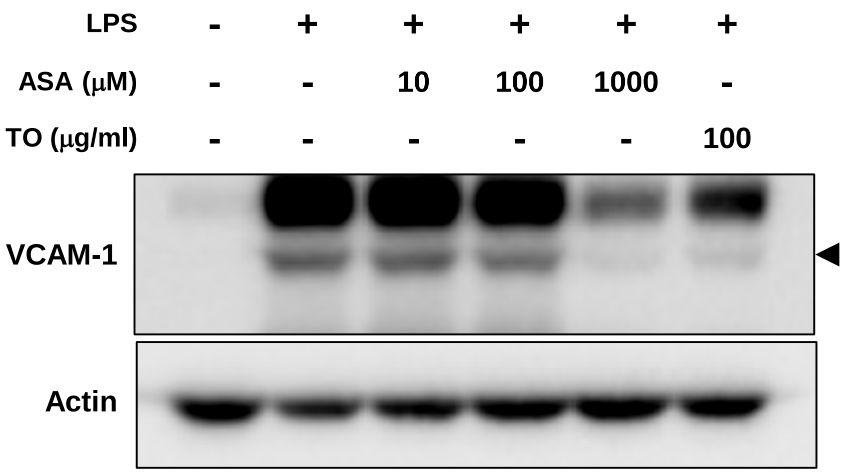

Additional file 1: Figure S1. Comparison of TO efficacy with acetylsalicylic acid (ASA). HU pretreated with TO (100 μg/ml) or different concentrations of ASA for one hour, and then stimulated with 1 μg/ml LPS for 24 h. VCAM-1 and actin levels were assessed by Western blot analysis. Arrow head indicates VCAM-1.

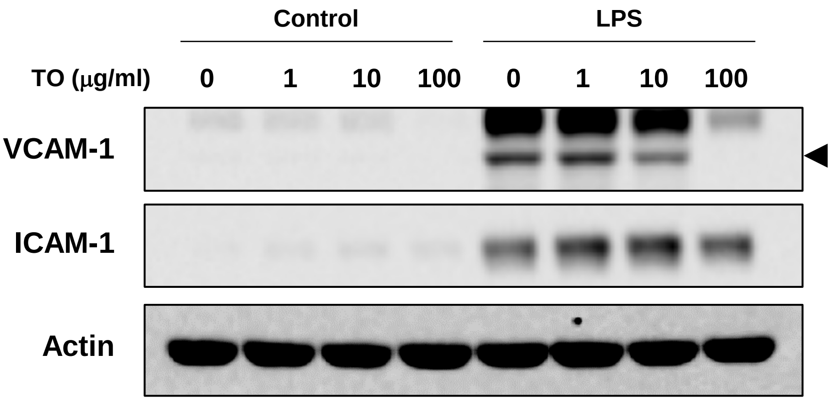

Additional file 1: Figure S2. TO decreases VCAM-1 induction dose dependently but does no levels in LPS-stimulated HUVECs. HUVECs were pretreated with different concentrations of TO for one hour, and then stimulated with 1 μg/ml LPS for 24 h. VCAM-1, ICAM-1, and actin levels were assessed by Western blot analysis. Arrow head indicates VCAM-1.

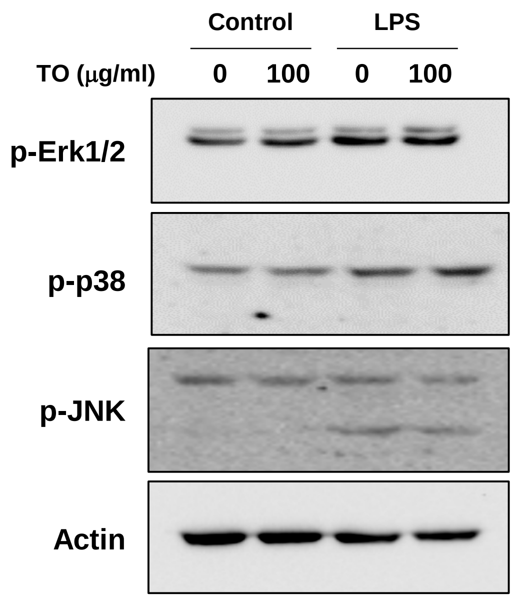

Additional file 1: Figure S3. TO does not affect MAPK activation induced by LPS stimulation
HUVECs were preincubated with TO (100 μg/ml) for one hour and then stimulated with LPS (1 μg/ml) for 30 min. MAPKs activation was assessed by Western blot analysis.
